# Evaluation of interventions to improve electronic health record documentation within the inpatient setting: a protocol for a systematic review

**DOI:** 10.1186/s13643-019-0971-2

**Published:** 2019-02-13

**Authors:** Lucia Otero Varela, Natalie Wiebe, Daniel J. Niven, Paul E. Ronksley, Nicolas Iragorri, Helen Lee Robertson, Hude Quan

**Affiliations:** 1 Department of Community Health Sciences, Cumming School of Medicine, University of Calgary, 3rd Floor TRW Building, 3280 Hospital Dr. NW, Calgary, AB T2N 4Z6 Canada; 2 Health Sciences Library, Libraries and Cultural Resources, University of Calgary, Calgary, Canada

**Keywords:** Electronic health records, Documentation, Quality improvement, Inpatient, Intervention, Systematic review protocol

## Abstract

**Background:**

Electronic health records (EHRs) are increasing in popularity across national and international healthcare systems. Despite their augmented availability and use, the quality of electronic health records is problematic. There are various reasons for poor documentation quality within the EHR, and efforts have been made to address these areas. Previous systematic reviews have assessed intervention effectiveness within the outpatient setting or within paper documentation. This systematic review aims to assess the effectiveness of different interventions seeking to improve EHR documentation within an inpatient setting.

**Methods:**

We will employ a comprehensive search strategy that encompasses four distinct themes: EHR, documentation, interventions, and study design. Four databases (MEDLINE, EMBASE, CENTRAL, and CINAHL) will be searched along with an in-depth examination of the grey literature and reference lists of relevant articles. A customized hybrid study quality assessment tool has been designed, integrating components of the Downs and Black and Newcastle-Ottawa Scales, into a REDCap data capture form to facilitate data extraction and analysis. Given the predicted high heterogeneity between studies, it may not be possible to standardize data for a quantitative comparison and meta-analysis. Thus, data will be synthesized in a narrative, semi-quantitative manner.

**Discussion:**

This review will summarize the current level of evidence on the effectiveness of interventions implemented to improve inpatient EHR documentation, which could ultimately enhance data quality in administrative health databases.

**Systematic review registration:**

PROSPERO CRD42017083494

**Electronic supplementary material:**

The online version of this article (10.1186/s13643-019-0971-2) contains supplementary material, which is available to authorized users.

## Background

**Fig. 1:**
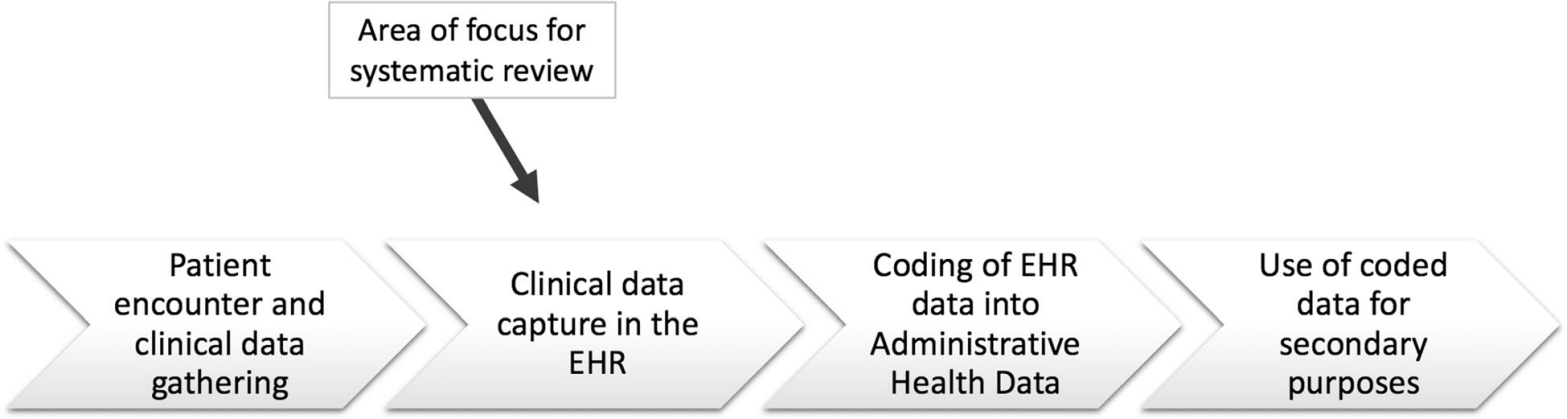
Data management chain and point of interest for EHR documentation quality improvement interventions

Healthcare professionals worldwide have transitioned from handwritten documentation to electronic reporting processes. In North America, over half of office-based practices and hospitals use some form of electronic health record (EHR) documentation [1]. Compared to conventional paper documentation, electronic health records produce clear, legible data that lends itself well to coders, computational analyses, and health service research. The administrative health record databases are fuelled by data produced by coders, who assign diagnostic codes to each diagnosis listed in patient charts. By removing the barrier of illegible or disorganized documentation, the quality of data in the administrative health record database is ameliorated. This data is then used for epidemiological studies, disease surveillance, and administrative and billing purposes [2]. Quality assurance of this data is thus crucial, and quality improvement strategies are being implemented at various points in the data management chain. As outlined in Fig. 1, this systematic review focuses on the quality of the data created during a clinical encounter, prior to its coding.

Because data quality is directly affected by the completion and accuracy of EHR documentation, it is important to assess and improve the quality of such documentation. Additionally, there has been a surge in research to improve EHR documentation due to the increase in medical errors associated with the use of EHRs [3]. Quality in EHRs may relate elements such as completeness, accuracy, clarity, and timeliness [4]. Although EHR documentation has existed since the 1960s, with the first computerized physician order entry system created in 1971 [5], a review of the medical literature reveals that the quality and usability of EHR documentation is generally poor [6]. Several problems with EHR documentation have been identified. These include structural problems where documentation quality suffers if the EHR system does not have built-in logic prohibiting the user from continuing onto the next section of documentation if the previous section has not been completed. Similarly, free-text fields, as opposed to point-and-click radio button documentation, have demonstrated increases in error [7]. Another common structural problem is the lack of standardization in EHR systems or vendors across all areas of healthcare delivery (i.e., outpatient versus inpatient EHR programs) [8]. In other instances, problems arise when EHR system users are not provided with adequate training and simply do not know how to use the system, leading to poor data quality. Resistance to EHR adoption further inhibits the standardization of documentation and can also impact data quality and usability [9].

Prior systematic reviews have explored ways to improve medical documentation; however, these were focused on the outpatient setting [10] or targeted EHR documentation of a specific EHR user [11]. Others failed to address electronic forms of documentation [12] or focused on a specific type of intervention to improve documentation [13, 14]. Noteworthy results from these systematic reviews illustrate the following: (1) a dearth of literature addressing EHR improvement, (2) successful interventions to improve EHR documentation (e.g., system add-ons, educational materials, and financial incentives), and (3) different indicators to measure quality of documentation, such as completeness and accuracy of patient information [10]. Using the PRISMA Protocol checklist, we outline our proposal for a systematic review of the literature to identify interventions, programs, or institutional changes (broadly referred to in this protocol as “interventions”) that have sought to improve EHR documentation in the inpatient setting and those that consequently may warrant implementation by EHR users [15].

### Focused questions

Accordingly, the questions we are addressing are:

1. What is the effectiveness of interventions seeking to improve inpatient hospital documentation in electronic health records?
  It should be noted that the word “seeking” is crucial to this question; studies will be included in the review if the intent of the intervention was to improve documentation quality, regardless of the study outcome.
2. What tools and metrics were used to measure the improvement in EHR documentation?

## Methods

### Search strategy

We will search the following databases: MEDLINE, EMBASE, Cochrane Central Register of Controlled Trials (CENTRAL), and CINAHL, with no language or date restrictions. Additionally, both investigators (LOV and NW) will complete a grey literature search, including conference proceedings identified through EMBASE. Experts in the field, identified from the review process, and other researchers who have previously worked on the topic will be contacted for further information about ongoing or unpublished studies. Reference lists of included studies will also be searched.

After consulting with two librarians, the search strategy was organized into four exhaustive themes, resulting in four Boolean searches using the term “or” to explode and search by keyword different subject headings:

1. Derivatives of “electronic health records” to specify the main outcome
2. Derivatives of “documentation” to refine the main outcome
3. Both general terms and specific examples of interventions, including synonyms or derivatives, to capture the vast array of interventions

Cochrane filter for randomized controlled trial (RCT), University of Alberta filter for observational studies, and PubMed Health filter for quasi-experimental studies to identify study designs [16–18]

Lastly, the Boolean operator “and” was used to combine the four search themes. An example of a detailed search strategy with all included terms is available in Additional file 1.

Improvement in documentation and its possible measures were not specified as search themes to avoid excluding studies that may have used an improvement measure not listed in the data extraction form. Further, since an intervention could be applied to the computer program or EHR “vendor,” rather than a human group of participants, EHR users were not specified in this search. An initial search in MEDLINE reveals approximately 1500 potential articles, with a similar result in EMBASE, indicating sufficient numbers to perform our analyses.

### Eligibility criteria and study selection

Detailed inclusion/exclusion criteria are outlined in Table 1. For the purpose of providing a comprehensive systematic review of the topic, we will not restrict this review to RCTs, but will incorporate all original literature reporting on the quality of EHR documentation. Consequently, experimental, quasi-experimental, and observational studies will be captured. The study population is primary users of inpatient EHRs, including physicians, nurses, diagnostic imaging staff, pharmacists, and clinical trainees (residents, interns, etc.). The interventions include but are not limited to activities, programs, or institutional changes applied to improve EHR documentation, such as the use of new software, dictation templates, audits, educational sessions, structured reporting, healthcare provider training, incentives, rewards, or penalties. Specifically, we will be looking for studies comparing interventions to controls (i.e., standard EHR documentation or a comparator intervention). The outcome of interest is improved EHR inpatient documentation, for which the measures have been identified from relevant literature and are further established by each individual study (Table 2).

**Table 1:** Inclusion and exclusion criteria for abstract and full-text screening stages of the systematic review

Criteria	Included	Excluded
Abstract screening		
Study design	Original research: observational, experimental, quasi-experimental	Letters, editorials, comments, book chapters, systematic reviews
Outcome	EHR documentation	Paper documentation Other studies unrelated to the topic: not looking at EHR nor documentation, animal studies
Setting	Inpatient or acute/care Single/multi-center	Outpatient, emergency department, clinic
Intervention	Variety of interventions	No intervention, only reporting on current documentation quality
Full-text screening		
Study design	Original research: observational, experimental, quasi-experimental	Letters, editorials, comments, book chapters, systematic reviews
Outcome	EHR documentation	Paper documentation Other studies unrelated to the topic: not looking at EHR nor documentation, animal studies
Setting	Inpatient or acute/care Single/multi-center	Outpatient, emergency department, clinic, family practice offices, minor/day/dental surgeries
Intervention	Variety of interventions	No intervention, only reporting on current documentation quality
Document type	Inpatient electronic records (authors contacted if unclear)	EHR implementation on paper-based system (unless study compared paper documentation to at least 2 other arms using electronic documentation) Not explicitly reporting on “inpatient” or “electronic”
Participants (EHR user)	Nurses, physicians, therapists, diagnostic imaging, pharmacists	Primary care providers (family physicians, general practitioners, etc.), researchers, coders, patients, management
Outcome	Improving EHR documentation (see Table 2)	Studies using EHR documentation to improve other healthcare service areas (e.g., patient care, healthcare delivery) or improved analytical features within EHR for research purposes. Clinical outcomes as primary or secondary goal

**Table 2:** Measures for “improved inpatient EHR documentation” and their definitions

Outcome measure	Definition
Medication accuracy	The absence of or decline in the number of errors and discrepancies present in the medication record
Document accuracy	The absence of or decline in the number of errors and discrepancies present in the EHR document
Completeness	The lack or decrease of missing information, as well as the addition of documented items within a medical record
Timeliness	A decrease in the time required to complete the document and also a shortening of the turnaround time necessary for the document to be available
Overall quality	Variously defined by each study and assessed through mean scores of personalized checklists or quality indicators
Clarity	A well-organized, readable, and easily understandable document
Length	The decrease in the number of lines or word count
Document capture	An increased number of documents created (not included in this review because of lack of data)
User satisfaction	Determined by the primary EHR users in surveys that evaluate their opinion on the implementation of the intervention

Both abstract and full-text screening phases will be done independently by two reviewers (LOV and NW) with the support of an eligibility criteria screening tool (Table 3). Titles and abstracts will be scanned to select articles for in-depth analysis. Articles will be selected for full-text review if both reviewers agree on eligibility criteria or if the abstract does not provide sufficient information to make a decision. Any discrepancies between reviewers will be discussed until an agreement is reached. When necessary, additional clarity regarding article eligibility will be requested by contacting the articles’ authors and examining unclear articles with another investigator (DJN). Inter-rater agreement will be assessed using the kappa statistic for both stages of screening.

**Table 3:** Eligibility criteria screening tool for use at the title, abstract, and full-text review screening stages

Inclusion/exclusion criteria for all screening stages: title, abstract, and the full-text (go from steps 1 to 6)	
1. Is the study conducted in humans? a. No—exclude b. Yes or uncertain—go to step 2	
2. Does the article represent an original study, including experimental, quasi-experimental, and observational study designs (e.g., no letters to the editor, book reviews, published study designs, or trial protocols)? a. No—exclude b. Yes or uncertain—go to step 3	
3. Does the study focus on electronic health records (EHRs)? a. No—exclude b. Yes or uncertain—go to step 4	
4. Does the article report on inpatient hospital data (e.g., no outpatient, emergency department, clinics)? a. No—exclude b. Yes or uncertain—go to step 5	
5. Is improvement in EHR documentation reported as an outcome? a. No—exclude b. Yes or uncertain—go to step 6	
6. Are any interventions being implemented to improve EHR documentation in the study? a. No—exclude b. Yes—include	
i. Full-text screening—continue in step 7	
Additional inclusion/exclusion criteria for full-text stage only	
7. Does this study reports on electronic records (i.e., explicitly mentioning “electronic” or derivatives, no paper documentation unless the study compared paper documentation to at least 2 other arms using electronic documentation)? a. No—exclude b. Yes or uncertain—go to step 8	
8. Is this study conducted within an inpatient setting (e.g., explicitly mentioning “inpatient” or derivatives, no outpatient, family practice offices, minor/day/dental surgeries)? a. No—exclude b. Yes or uncertain—go to step 9	
9. Are the users of the EHR nursing staff, pharmacists, diagnostic imaging staff, physicians, respiratory therapists (e.g., no researchers, primary care providers, coding specialists)? a. No—exclude b. Yes or uncertain—go to step 10	
10. Does this study implement an intervention aiming at improving EHR documentation (i.e., no studies without intervention or only report on current documentation quality)? a. No—exclude b. Yes or uncertain—include	

### Data extraction and study quality assessment tool

REDCap was used to create a data extraction form with built-in logic to collect pertinent information from all included studies [19], available in Additional file 2. This logic also comprises hidden questions that appear when a certain answer is chosen. This feature is a “real-time” function that cannot be depicted in the printed form embedded as an additional file. The form focuses on the detailed study characteristics (e.g., EHR users, type of setting, outcome measures). For results of interventions, the reviewers will extract differences between intervention groups, as well as before and after, or cross-sectional designs. Results will be expressed as means, medians, proportions, or effect size, depending on the design. The data extraction tool will also allow reviewers to abstract the measure used to identify high- or low-quality EHR documentation. Study quality and systematic error (bias) will be assessed using a hybrid of the Downs and Black Scale and the Newcastle-Ottawa Scale, including 11 items to encompass experimental, quasi-experimental, and observational study designs (Additional file 3) [20, 21].

### Data synthesis/analysis

Given the expected heterogeneity in methods and possibly in results in the extant literature, it is unclear if meta-analysis will be possible. We will explore the factors associated with heterogeneity and attempt to assess the effect of a number of variables on the results of the identified intervention. These variables include but are not limited to the type of EHR user (physician, nurse, pharmacist, therapist, etc.), type of setting (urban or rural), size of setting (single or multi-center) area of clinical practice, demographic characteristics of users, and experience with EHR (years of use). The final number and the characteristics of studies identified for inclusion in and exclusion from the systematic review will be reported in a PRISMA flow diagram. We will tabulate all extracted data, including participant characteristics, study designs, interventions, instruments, and study results. For the primary question (overall effectiveness of interventions), we will describe the range of results obtained across all studies, grouped by intervention. Differences between study results will be presented in a narrative form with semi-quantitative analysis, unless meta-analyses are feasible, as outlined above. To address the secondary question, we will describe the tools used to identify interventions with high or low effectiveness.

## Discussion

To our knowledge, this will be the first systematic review to identify and evaluate interventions that are specifically aimed to improve EHR documentation in an inpatient setting.

The strengths of this systematic review include an in-depth search strategy, organized into four comprehensive themes; an elaborated eligibility criteria; and an adapted study quality assessment tool. Thus, this protocol provides a methodologically rigorous template for future similar systematic or scoping review studies for effective interventions.

Outcomes of this study will be applicable to clinicians, policy-makers, health information managers, quality improvement specialists, and coding organizations and will provide a direction for future researchers seeking to improve administrative discharge database quality.

## Additional files

Additional file 1: Search strategy for MEDLINE database. Accessed on November 8, 2017. (PDF 329 kb)

Additional file 2: REDCap data extraction form. Data extraction form with built-in logic created with REDCap and used to collect relevant information from all included studies. (PDF 2243 kb)

Additional file 3: Study quality assessment tool adapted from Downs and Black Scale combined with Newcastle-Ottawa Scale (NOS). Eleven-item hybrid of Downs and Black and Newcastle-Ottawa Scales, used to assess study quality and systematic error (bias) of selected studies, encompassing experimental, quasi-experimental, and observational study designs. (PDF 644 kb)

## Abbreviations

CENTRAL: Cochrane Central Register of Controlled Trials
CINAHL: Cumulative Index to Nursing and Allied Health Literature
EHR: Electronic health record
EMBASE: Excerpta Medica dataBASE
MEDLINE: Medical Literature Analysis and Retrieval System Online
PRISMA: Preferred Reporting Items for Systematic Reviews and Meta-Analyses
RCT: Randomized controlled trial
REDCap: Research Electronic Data Capture
