# Supplementary material for: Evaluation of interventions to improve electronic health record documentation within the inpatient setting: a protocol for a systematic review
Source: Syst Rev. 2019 Feb 13;8:54. doi: 10.1186/s13643-019-0971-2 (PMC6373133; doi:10.1186/s13643-019-0971-2)
Supplement: Supplementary file 1 — Search strategy for MEDLINE database. Accessed on November 8, 2017. (PDF 329 kb) [file 13643_2019_971_MOESM1_ESM.pdf]

Additional file 1. Search strategy for MEDLINE database. Accessed on November 8th, 2017.

1 Electronic Health Records/ or Medical Records Systems, Computerized/ or Medical Records/ or hospital records/ or nursing records/ (109025)
2 ("electronic patient record*" or "electronic medical record*" or "electronic health record*" or "computeri#ed patient record*" or "computeri#ed medical record*" or "computeri#ed health record*" or "ambulatory medical record*" or EHR or AMR or EPR).tw. (48059)
3 exp Medical Records Systems, Computerized/ (35997)
4 1 or 2 or 3 (146360)
5 terminology as topic/ or "forms and records control"/ or "abstracting and indexing as topic"/ or coding, clinical/
or documentation/ or data collection/ (175234)
6 (controlled vocabulary or controlled language or controlled terminology or controlled coding or controlled documentation or data collection).tw. (64518)
7 exp documentation/ or documentation*.tw. or "EHR documentation*".tw. (1003713)
8 5 or 6 or 7 (1207641)
9 exp Algorithms/ or template*.tw. (365715)
10 ("structured reporting" or "point and click").tw. (458)
11 ("mandatory field*" or "required field* mandatory text*" or "required text*" or "vendor format*").tw. (60)
12 exp Speech Recognition Software/ or "dictation template*".tw. (654)
13 ("incentiv*" or "decentiv*").tw. or exp Motivation/ or "reward*".tw. or "compensat*".tw. or "punishment*".tw. (374300)
14 audit*.tw. (136634)
15 feedback*.tw. (122428)
16 ("reminder*" or "reinforce*").tw. (92088)
17 ("education* session*" or "train*" or "workshop*" or "seminar*" or "lecture*" or "in-service" or inservice or "on-site" or onsite).tw. (548252)
18 (intervention* or improv* or program* or participat* or involv* or initiat* or performance*).tw. (6240403)
19 9 or 10 or 11 or 12 or 13 or 14 or 15 or 16 or 17 or 18 (7090724)
20 exp Randomized Controlled Trials as Topic/ or (randomized controlled trial or controlled clinical trial).pt. or randomized.ab. or placebo.ab. or clinical trials as topic.sh. or randomly.ab. or trial.ti. (1292743)
21 exp Observational Study/ or Epidemiologic studies/ or cross sectional.tw. or cross-sectional studies/ or exp cohort studies/ or cohort$.tw. or exp case-control studies/ or (case* and control*).tw. or (case* and series).tw. or controlled clinical trial.pt. (3046564)
22 (nonequivalent control group or posttesting or pretesting or pretest posttest design or pretest posttest control group design or quasi experimental methods or quasi experimental study or time series or time series analysis).sh. (0)
23 (((nonequivalent or non equivalent) adj3 control$) or posttest$ or post test$ or pre test$ or pretest$ or quasi experiment$ or quasiexperiment$ or timeseries or time series).tw. (66074)
24 exp animals/ not humans.sh. (4743197)
25 20 or 21 or 22 or 23 (3999839)
26 25 not 24 (3830229)
27 4 and 8 and 19 and 26 (2593)
