# Supplementary material for: Evaluation of interventions to improve electronic health record documentation within the inpatient setting: a protocol for a systematic review
Source: Syst Rev. 2019 Feb 13;8:54. doi: 10.1186/s13643-019-0971-2 (PMC6373133; doi:10.1186/s13643-019-0971-2)
Supplement: Supplementary file 2 — REDCap data extraction form. Data extraction form with built-in logic created with REDCap and used to collect relevant information from all included studies. (PDF 2243 kb) [file 13643_2019_971_MOESM2_ESM.pdf]

Additional file 2. REDCap data extraction form

Reviewer Initials ______________________________

Study ID ______________________________

Author ______________________________
(Last name of author)

Year ______________________________
(Year of publication)

Study Characteristics

What continent was the study conducted in?

- ◯ North America
- ◯ South America
- ◯ Europe
- ◯ Asia
- ◯ Africa
- ◯ Australia/Oceania

What country was the study conducted in?

- ◯ Canada
- ◯ U.S.A.
- ◯ Mexico
- ◯ Germany
- ◯ France
- ◯ Italy
- ◯ Japan
- ◯ China
- ◯ North/South Korea
- ◯ Australia
- ◯ New Zealand
- ◯ Brazil
- ◯ Russia
- ◯ Other

Please specify the country. ______________________________

What type of hospital setting was the study conducted in? Select all that apply.

- ☐ Community hospital
- ☐ Teaching hospital
- ☐ Academic hospital
- ☐ General hospital
- ☐ Specialty care hospital
- ☐ Urban hospital setting
- ☐ Rural hospital setting
- ☐ Single centre
- ☐ Multi centre

What specialty area was the study conducted in? Select all that apply.

- ☐ Trauma
- ☐ Intensive Care
- ☐ Renal
- ☐ Cardiac
- ☐ Neurological
- ☐ Orthopaedic
- ☐ Hematological
- ☐ Paediatric
- ☐ Other
- ☐ All of the above

Who was the intervention applied on? (In other words, who were the study participants?) Select all that apply.

- ☐ Nurses
- ☐ Physicians
- ☐ Pharmacists
- ☐ Occupational Therapists
- ☐ Radiologists
- ☐ Technicians
- ☐ Respiratory Therapists
- ☐ Students
- ☐ None of the above

How many study participants were there?	______________________________<br>(Enter '0' if not provided. Enter '.' if not applicable.)
What was the female proportion of participants?	______________________________<br>(%)
How many records were used?	______________________________<br>(Enter '0' if not provided. Enter '.' if not applicable.)
What intervention was implemented to improve EHR documentation? Select all that apply.	☐ New EHR reporting system (e.g. structured, point-and-click, stroke-specific, proforma)<br>☐ New EHR software or vendor<br>☐ New EHR modality (e.g. hand-held computer, laptop)<br>☐ Reminders within EHR system<br>☐ Template/Guideline/Example displayed to EHR user<br>☐ Educational program (e.g. training session, in-service, video, simulation)<br>☐ Feedback provided to EHR user<br>☐ Audit<br>☐ Other
Please describe the intervention used.	______________________________________
What was the study design?	◯ RCT<br>◯ Observational<br>◯ Quasi-Experimental
Please specify. Select all that apply.	☐ Prospective<br>☐ Retrospective<br>☐ Pre-test/Post-test<br>☐ Interrupted time-series<br>☐ Combination<br>☐ Other<br>☐ Not specified
What was the intervention compared to?	☐ Standardized documentation<br>☐ Dictation<br>☐ Non-structured reporting<br>☐ No template provided<br>☐ Paper documentation<br>☐ No education provided<br>☐ Baseline (no further description)
What was the intervention compared to? Select all that apply.	☐ Standardized documentation<br>☐ Dictation<br>☐ Non-structured reporting<br>☐ No template provided<br>☐ No education provided<br>☐ Paper documentation<br>☐ Baseline (no further description)
Please select the type of observational study. Select all that apply.	☐ Prospective cohort<br>☐ Retrospective cohort<br>☐ Prospective case-control<br>☐ Retrospective case-control<br>☐ Cross-sectional<br>☐ Unsure

What were the control and intervention arms?

____

Was randomization used?

- ◯ No
- ◯ Yes
- ◯ Not specified
- ◯ Unsure

Was there assessment for bias?

- ◯ No
- ◯ Yes
- ◯ Not specified
- ◯ Unsure

Was blinding used?

- ◯ No
- ◯ Yes
- ◯ Not specified
- ◯ Unsure

Was allocation concealment used?

- ◯ No
- ◯ Yes
- ◯ Not specified
- ◯ Unsure

Was EHR documentation improved?

- ◯ No, it worsened
- ◯ Yes
- ◯ Yes and No
- ◯ No, it stayed the same

How was the intervention measured? Select all that apply.

- ☐ Medication accuracy (e.g. med. errors/reconciliation)
- ☐ Timeliness (e.g. time to complete documentation, time to availability of documentation)
- ☐ Completeness (missing information in document)
- ☐ Capture of documentation (more or less reports)
- ☐ Documentation accuracy (e.g. errors, discrepancies)
- ☐ Quality (e.g. quality indicators, overall quality)
- ☐ Clarity (as judged by reviewer of document)
- ☐ Length
- ☐ Other

Please describe the intervention measure.

____

Was documentation improved according to medication accuracy?

- ◯ No, it was worse than control group
- ◯ No, it stayed the same
- ◯ Yes, it improved
- ◯ Unsure

Was documentation improved according to timeliness?

- ◯ No, it was worse than control group
- ◯ No, it stayed the same
- ◯ Yes, it improved
- ◯ Unsure

Was documentation improved according to completeness?

- ◯ No, it was worse than control group
- ◯ No, it stayed the same
- ◯ Yes, it improved
- ◯ Unsure

Was documentation improved according to capture of documentation?

- ◯ No, it was worse than control group
- ◯ No, it stayed the same
- ◯ Yes, it improved
- ◯ Unsure

Was documentation improved according to documentation accuracy?	◯ No, it was worse than control group<br>◯ No, it stayed the same<br>◯ Yes, it improved<br>◯ Unsure
Was documentation improved according to quality?	◯ No, it was worse than control group<br>◯ No, it stayed the same<br>◯ Yes, it improved<br>◯ Unsure
Was documentation improved according to clarity?	◯ No, it was worse than control group<br>◯ No, it stayed the same<br>◯ Yes, it improved<br>◯ Unsure
Was documentation improved according to length?	◯ No, it was worse than control group<br>◯ No, it stayed the same<br>◯ Yes, it improved<br>◯ Unsure
Was documentation improved according to this other measure?	◯ No, it was worse than control group<br>◯ No, it stayed the same<br>◯ Yes, it improved<br>◯ Unsure
What quantity of improvement was reported for medication accuracy?	________________________________<br>(Specify if mean, rate, improved, worsened, etc.)
What quantity of improvement was reported for timeliness?	________________________________<br>(Specify if mean, rate, improved, worsened, etc.)
What quantity of improvement was reported for completeness?	________________________________<br>(Specify if mean, rate, improved, worsened, etc.)
What quantity of improvement was reported for capture of documentation?	________________________________<br>(Specify if mean, rate, improved, worsened, etc.)
What quantity of improvement was reported for documentation accuracy?	________________________________<br>(Specify if mean, rate, improved, worsened, etc.)
What quantity of improvement was reported for quality?	________________________________<br>(Specify if mean, rate, improved, worsened, etc.)
What quantity of improvement was reported for clarity?	________________________________<br>(Specify if mean, rate, improved, worsened, etc.)
What quantity of improvement was reported for length?	________________________________<br>(Specify if mean, rate, improved, worsened, etc.)
Please provide a p-value for medication accuracy, if available.	________________________________
Please provide a p-value for timeliness, if available.	________________________________
Please provide a p-value for completeness, if available.	________________________________
Please provide a p-value for capture of documentation, if available.	________________________________
Please provide a p-value for accuracy, if available.	________________________________
Please provide a p-value for quality, if available.	________________________________

Please provide a p-value for clarity, if available.	______________________
Please provide a p-value for length, if available.	______________________
Please provide a p-value for this other measure, if available.	______________________
Was there a difference in EHR documentation between study participants?	◯ No<br>◯ Yes
What factor influenced the difference in documentation between study participants? Select all that apply.	☐ EHR documentation setting<br>☐ Student versus non-student<br>☐ Years of experience<br>☐ Age<br>☐ Gender
Were study participants followed-up?	◯ No<br>◯ Yes<br>◯ Unsure
For how long?	______________________<br>(Please specify unit (days, weeks, months, years).)
