# Supplementary material for: Evaluation of interventions to improve electronic health record documentation within the inpatient setting: a protocol for a systematic review
Source: Syst Rev. 2019 Feb 13;8:54. doi: 10.1186/s13643-019-0971-2 (PMC6373133; doi:10.1186/s13643-019-0971-2)
Supplement: Supplementary file 3 — Study quality assessment tool adapted from Downs and Black Scale combined with Newcastle-Ottawa Scale (NOS). Eleven-item hybrid of Downs and Black and Newcastle-Ottawa Scales, used to assess study quality and systematic error (bias) of selected studies, encompassing experimental, quasi-experimental, and observational study designs. (PDF 644 kb) [file 13643_2019_971_MOESM3_ESM.pdf]

Additional file 3. Study quality assessment tool adapted from Downs and Black combined with Newcastle-Ottawa Scale (NOS).

	Yes	No	Unable to determine
Reporting			
1. Was the research question or objective in this paper clearly stated?			
2. Was the study population clearly specified and defined?			
3. Are the interventions of interest clearly described?			
4. Are the main findings of the study clearly described?			
5. Have the negative and positive effects been reported?			
External validity			
6. Were the subjects asked to participate in the study representative of the entire population from which they were recruited?			
7. Were the places and facilities where the interventions were implemented representative of the setting for the majority of EHR usage?			
Internal validity – bias			
8. Were the outcome measures clearly defined, valid, reliable, and implemented consistently across all study participants?			
9. Were the statistical tests used to assess the main outcomes appropriate?			
Internal validity – confounding (selection bias)			
10. Were the participants in different intervention groups recruited from the same population?			
11. Were inclusion and exclusion criteria for being in the study pre-specified and applied uniformly to all participants?			
